# Molecular detection and genetic characterization of small rodents associated *Bartonella* species in Zhongtiao Mountain, China

**DOI:** 10.1371/journal.pone.0264591

**Published:** 2022-02-28

**Authors:** Juan Yu, Xiong-Ying Zhang, Yun-Xia Chen, Hong-Bing Cheng, Dong-Mei Li, Hua-Xiang Rao

**Affiliations:** 1 Department of Basic Medical Sciences, Changzhi Medical College, Changzhi, Shanxi, China; 2 State Key Laboratory for Infectious Disease Prevention and Control, Collaborative Innovation Center for Diagnosis and Treatment of Infectious Diseases, National Institute for Communicable Disease Control and Prevention, Chinese Center for Disease Control and Prevention, Beijing, China; 3 Department of Public Health and Preventive Medicine, Changzhi Medical College, Changzhi, Shanxi, China; University of Bari, ITALY

## Abstract

The prevalence and molecular characteristics of *Bartonella* infections in small rodents in the Zhongtiao Mountain, China have been explored. In this study, the liver, spleen and kidney tissues of captured rodents were used for *Bartonella spp*. detection and identification by combination of real-time PCR of transfer-mRNA (*ssrA*) gene and traditional PCR and sequencing of citrate synthase (*gltA*) gene. It was shown that 49.52% of the rodents (52/105) were positive for *Bartonella spp*.. The infection rate in different gender (*χ*^2^ = 0.079, *P* = 0.778) and tissues (*χ*^2^ = 0.233, *P* = 0.890) of small rodents did not have statistical difference, but that in different small rodents (Fisher’s exact test, *P* < 0.001) and habitats (*χ*^2^ = 5.483, *P* = 0.019) had statistical difference. And, the sequencing data suggests that *Bartonella* sequences (n = 31) were identified into three species, including 14 of *B*. *grahamii*, 3 of *B*. *queenslandensis* and 14 of *unknown Bartonella* species. Phylogenetic analysis showed that *B*. *grahamii* sequences were clustered with the isolates from South Korea and China, and *B*. *queenslandensis* sequences were mainly closely related to the isolates from China and Thailand. The genetic diversity analysis showed that *B*. *grahamii* and *B*. *queenslandensis* sequences exhibited noticeable intraspecies diversity. Taken together our data demonstrates the high prevalence and genetic diversity of *Bartonella* infections in small rodents in the Zhongtiao Mountain, especially a potential novel *Bartonella* specie was detected, which could benefit the prevention and control of rodent-*Bartonella* species in this area.

## Introduction

*Bartonella* species are an emerging family of vector-borne, facultative, gram-negative, haemotrophic bacteria, known to infect mammalian erythrocytes and endothelial cells and might cause human Bartonellosis [1]. There are currently over 40 species of *Bartonella* identified, and 14 of them are known to be zoonotic [2]. They have a wide range of reservoirs, including cats, dogs, rodents, bats, and so on [3]. Humans can be infected by close contact with rodents, cats and dogs, including typical and common diseases, such as cat scratch disease [4] and trench fever [5], and atypical diseases, such as neuroretinitis, arthritis, endocarditis, myocarditis, osteomyelitis, bacteremia, etc [6–8].

Since *Bartonella* was first isolated from HIV patients in 1993 [9], more than 10 *Bartonella* species have been recognized as human pathogens, such as *B*. *bacilliformis* [10], *B*. *quintana* [11], *B*. *henselae* [12], *B*. *elizabethae* [13], *B*. *clarridgeiae* [14], *B*. *koehlerae* [15], *B*. *vinsonii* subsp. *Arupensis* [16], *B*. *vinsonii* subsp. *Berkhoffii* [17], *B*. *grahamii* [18, 19], *B*. *rochalimae* [20], *B*. *tamiae* [21], *B*. *ancashensis* [22], *B*. *washoensis* [23], and the first three species are reportedly responsible for the majority of clinical illness [24]. In recent years, an increasing number of studies on *Bartonella* have been carried out successively in various countries around the world, including the United States [25], Europe [26], Asia [27, 28], Africa [29], Latin America [30] and Oceania [31]. These studies also reveal that the prevalence of *Bartonella* in rodents varies greatly in different countries and regions, making it necessary to investigate the rodents-associated *Bartonella* infection in different areas.

The Zhongtiao Mountain, is one of the major mountains, in the southern Shanxi Province of China, with an average elevation of 1200–2300 m, spread across the three cities of Linfen, Yuncheng and Jincheng. Here, we selected Pingquan Village within the Yangcheng County of Jincheng City as the rodents trapping site, as it is located at the east of Zhongtiao Mountain, and 30 km from the Manghe National Nature Reserve. Pingquan Village, as the Red Tourist Spot, with the development of tourism, the direct or indirect contact between humans and rodents increased, and the risk of transmission of rodents-associated *Bartonella* infection also increased. However, investigations of *Bartonella* species in small rodents in this area have not been reported. Given this, this study was designed to explore the prevalence and genetic diversity of *Bartonella* species in small rodents in the parts of Zhongtiao Mountain, in an effort to provide the necessary scientific information to help support the creation of a plan for the control and prevention of *Bartonella* infection in humans in this area.

## Materials and methods

### Ethical statement

This study was approved by the Ethics Committee of Changzhi Medical College (No: DW2021052). All animals were treated according to the Guidelines of Regulations for the Administration of Laboratory Animals (Decree No. 2 of the State Science and Technology Commission of the People’s Republic of China, 1988) and the Guidelines for Treating Animals Kindly from Ministry of Science and Technology of the People’s Republic of China. All efforts were made to minimize discomfort to the animals.

### Rodents collection

Small rodents were captured using snap traps from Pingquan Village (35.36° N, 112.32° E) within Yangcheng County of Shanxi Province during May 2021. The trapped rodents were identified by morphology and DNA barcoding via the Cytochrome C oxidase subunit I (COI) gene. The liver, spleen and kidney tissues were then harvested under sterile conditions from each rodent after euthanasia, and stored at -80 °C until use.

### *Bartonella* detection

DNA was extracted from approximately 10 mg of each liver, spleen and kidney tissues using the TIANamp Micro DNA Kit (TIANGEN Biotech (Beijing) Co., Ltd., China) as directed by the manufacturer. Real-time PCR was performed to detect the *Bartonella* transfer-mRNA (*ssrA)* gene. DNA amplification was performed in 20 μL mixtures containing 10 μL HR qPCR Master Mix (Shanghai Huirui Bio-Tech Co., Ltd., Shanghai, China), 5 μL double-distilled H_2_O, 0.8 μL (10 μmol/L) of each primer and 0.4 μL (10 μmol/L) probe (*ssrA*-F: GCTATGGTAATAAATGGACAATGAAA TAA; *ssrA*-R: GCTTCTGTTGCCAGGTG; *ssrA*-P: FAM-ACCCCGCTTAAACCTG CGACG-BHQ1) [32] and 3 μL DNA Template. *ssrA* amplification was performed under the following conditions: one cycle for 5 min at 95 °C; 40 cycles for 15 s at 95 °C, 45 s at 60 °C, and positive and negative control were set.

### *Bartonella* sequencing

For *ssrA* gene positive samples, *Bartonella* citrate synthase (*gltA*) gene amplification was further performed. DNA amplification was performed according to the manufacture’s protocols of TaKaRa PCR Amplification Kit (Takara Bio Inc., Japan) in 20 μL mixtures containing 2 μL 10 × PCR buffer, 1.6 μL dNTP mix, 0.1 μL Taq, 13.5 μL double-distilled H_2_O, 0.4 μL (10 μmol/L) of each primer (BhCS781.p: GGGG ACCAGCTCATGGTGG; BhCS1137.n: AATGCAAAAAGAACAGTAAACA [33]), and 2 μL of DNA template. *gltA* amplification was performed under the following conditions: one cycle for 5 min at 94 °C; 35 cycles for 30 s at 94 °C, 30 s at 55 °C, and 60 s at 72 °C; and a final extension for 10 min at 72 °C. Next, PCR products were identified by 1.5% agarose gel electrophoresis, and then sent to Shanghai BioGerm Medical Technology Co., Ltd (Shanghai, China) for sequencing.

### Phylogenetic analysis

The sequences generated in this study were submitted to the GenBank (accession numbers: MZ672181-MZ672211). The nucleotide sequence homology was blasted against reported *Bartonella* species sequences in the GenBank using the BLAST program available from the National Center for Biotechnology Information website (http://blast.ncbi.nlm.nih.gov/Blast.cgi). Besides, the *gltA* sequences of *B*. *grahamii* and *B*. *queenslandensis* in GenBank released before July 2021 were collected for traceability analysis of these two *Bartonella* species in our study. For the sequences isolated of the same strain from the same host in the same laboratory at the same time, we randomly selected a sequence as the reference. Phylogenetic tree was created using the maximum-likelihood method with MEGA version 7.0, and bootstrap values were calculated with 1000 replicates [34, 35]. *Brucella abortus* was used as the outgroup.

### Genetic diversity analysis

The polymorphism of nucleotide sequences, including the number of polymorphic sites (S), the number of haplotypes (H), the nucleotide diversity (π), the average number of nucleotide differences (*k*) and the haplotype diversity (Hd), were analyzed using DNASP 5.10 software. We used a sliding window interval of 25 bp to determine which segment of the target gene sequence had the highest nucleotide diversity (π) by analyzing 100 bp at a time across the length of the gene.

### Statistical analysis

The positive rates of *Bartonella spp*. in different genders, tissues, and habitats of small rodents were analyzed using the chi-square test. The positive rates of *Bartonella* in different rodents were analyzed using Fisher’s exact probability method. All data were analyzed using SPSS 22.0 (SPSS, Inc., Chicago, IL, USA). *P* < 0.05 was considered statistically significant.

## Results

### Animal collection

In total, 105 small rodents were captured and identified into eight species, including *Apodemus agrarius* (32), *Eothenomys inez* (28), *Apodemus draco* (19), *Mus musculus* (14), *Niviventer confucianus* (9), *Apodemus peninsulae* (1), *Rattus tanezumi* (1), and *Tscherskia triton* (1).

### *Bartonella* infections

In total, 52 small rodents were positive for *Bartonella* infection by qPCR, with an infection rate of 49.52% (52/105). The animals were classified into six species (*A*. *agrarius* (26/32), *E*. *inez* (10/28), *A*. *draco* (5/19), *M*. *musculus* (1/14), *N*. *confucianus* (9/9), *A*. *peninsulae* (1/1)), and the difference of positive rate among these species was shown to be statistically significant (*P <* 0.001, Fisher’s exact test) (Table 1). Unfortunately, during tissue collection we missed one of the spleen samples, the positive rates for the remaining samples in the liver, spleen and kidney were 46.67% (49/105), 48.08% (50/104) and 49.52% (52/105) respectively, and statistical evaluation confirmed that there were no significant differences in positive rate between these tissues (*χ*^2^ = 0.233, *P* = 0.890) (Table 1).

**Table 1:** Positive rate of *Bartonella* infection in different tissues of small rodents.

Host	Liver		Spleen		Kidney		Total	
	No. detection	No. PCR positive (%)	No. detection	No. PCR positive (%)	No. detection	No. PCR positive (%)	No. captured	No. PCR positive (%)
AA	32	26 (81.25)	31	25 (80.65)	32	23 (71.88)	32	26 (81.25)
EI	28	9 (32.14)	28	10 (35.71)	28	8 (28.57)	28	10 (35.71)
AD	19	4 (21.05)	19	5 (26.32)	19	5 (26.32)	19	5 (26.32)
MM	14	0 (0.00)	14	0 (0.00)	14	1 (7.14)	14	1 (7.14)
NC	9	9 (100.00)	9	9 (100.00)	9	9 (100.00)	9	9 (100.00)
AP	1	1 (100.00)	1	1 (100.00)	1	1 (100.00)	1	1 (100.00)
RT	1	0 (0.00)	1	0 (0.00)	1	0 (0.00)	1	0 (0.00)
TT	1	0 (0.00)	1	0 (0.00)	1	0 (0.00)	1	0 (0.00)
Total	105	49 (46.67)	104	50 (48.08)	105	47 (44.76)	105	52 (49.52)

AA: *Apodemus agrarius*, EI: *Eothenomys inez*, AD: *Apodemus draco*, MM: *Mus musculus*, NC: *Niviventer confucianus*, AP: *Apodemus peninsulae*, RT: *Rattus tanezumi*, TT: *Tscherskia triton*.

Of the 105 small rodents, 45 were males, 60 were females, and the positive rate was 51.11% (23/45) in males and 48.33% (29/60) in females, which was not statistically significant (*χ*^2^ = 0.079, *P* = 0.778). There were 17 small rodents from three species captured in villages, with a *Bartonella* infection rate of 23.53% (4/17). 88 small rodents from seven species were captured in the returning farmland to forests (RFF), with an infection rate of 54.55% (48/88). Thus, the *Bartonella* infections rates in the rodents from different habitats were significantly different (*χ*^2^ = 5.483, *P* = 0.019) (Table 2).

**Table 2:** Positive rate of *Bartonella* infection of small rodents in different habitats.

Habitats	Host								No. captured	No. PCR positive	Positive rate (%)
	AA	EI	AD	MM	NC	AP	RT	TT			
Village	0	0	0	13	3	0	1	0	17	4	23.53
RFF	32	28	19	1	6	1	0	1	88	48	54.55
Total	32	28	19	14	9	1	1	1	105	52	49.52

RRF: Returning farmland to forests.

### *Bartonella* identifications

**Fig 1:**
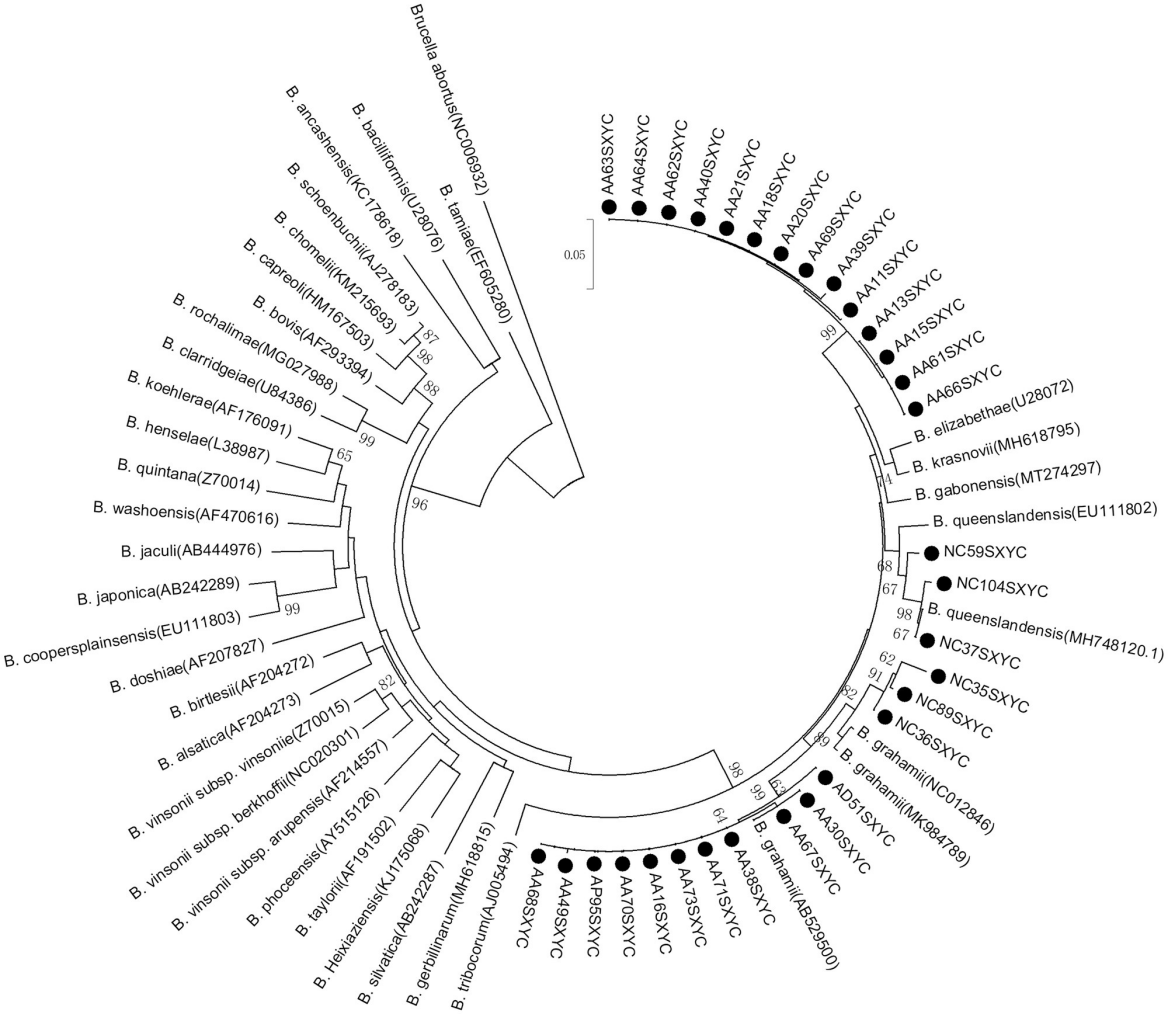
Phylogenetic trees constructed based on *gltA* gene of 31 *Bartonella* sequences obtained from the Zhongtiao Mountain.

**Fig 2:**
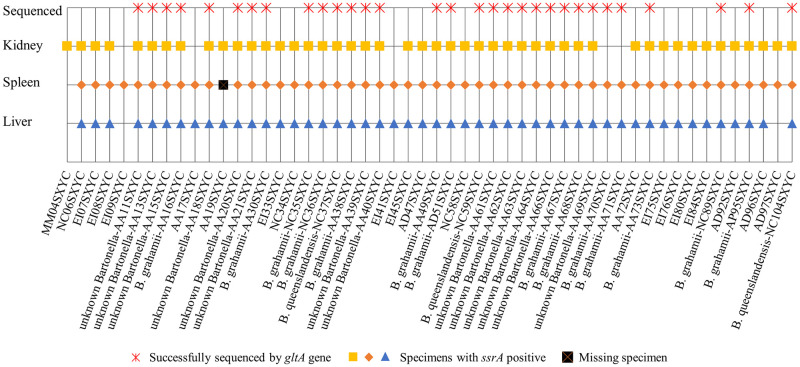
Detection of *Bartonella* species in various tissues of naturally infected rodents.

Of the 53 *gltA* sequences identified from 31 animals positive for *Bartonella*, 18 small rodents (58.06%) had the sequences in more than two tissues. The DNA sequence homology and phylogenetic analyses of the *gltA* gene indicated that three *Bartonella* species were detected in the liver, spleen and kidney of these small rodents, and the *Bartonella* species detected in different tissues of each small rodents were consistent. It was shown that 14 sequences were *B*. *grahamii* with 96.32–99.68% identity, including nine sequences from *A*. *agrarius*, three sequences from *N*. *confucianus*, one sequence from *A*. *draco* and one sequence from *A*. *peninsulae*. Three sequences from *N*. *confucianus* were *B*. *queenslandensis* with 97.94–99.42% identity. And, 14 sequences from *A*. *agrarius* were *unknown Bartonella* species, which shared 94.49–95.69% nucleotide sequence similarity in their *gltA* fragment with the nearest species of *Bartonella*, *B*. *krasnovii*, *B*. *gabonensis* and *B*. *elizabethae*, respectively (Figs 1 and 2).

In addition, 23 *Bartonella* sequences of two different species were obtained from *A*. *agrarius*, including nine *B*. *grahamii* and 14 *unknown Bartonella* species. Six *Bartonella* sequences of two different species were obtained from *N*. *confucianus*, including three *B*. *grahamii* and three *B*. *queenslandensis*. Another two *B*. *grahamii* sequences were obtained from *A*. *draco* and *A*. *peninsulae*, and there were no *Bartonella* sequences was identified in *E*. *inez*, *M*. *musculus*, *R*. *tanezumi* and *T*. *triton* (Table 3). It indicated that *A*. *agrarius* may be more susceptible to *Bartonella* than other rodents in this study, and *Bartonella* infection displays some degree of host specificity.

**Table 3:** Distribution of *Bartonella* species in the infected small rodents.

Host	*B*. *grahamii*	*B*. *queenslandensis*	*unknown Bartonella*	Total
AA	9	0	14	23
EI	0	0	0	0
AD	1	0	0	1
MM	0	0	0	0
NC	3	3	0	6
AP	1	0	0	1
RT	0	0	0	0
TT	0	0	0	0
Total	14	3	14	31

### *Bartonella* tracing

**Fig 3:**
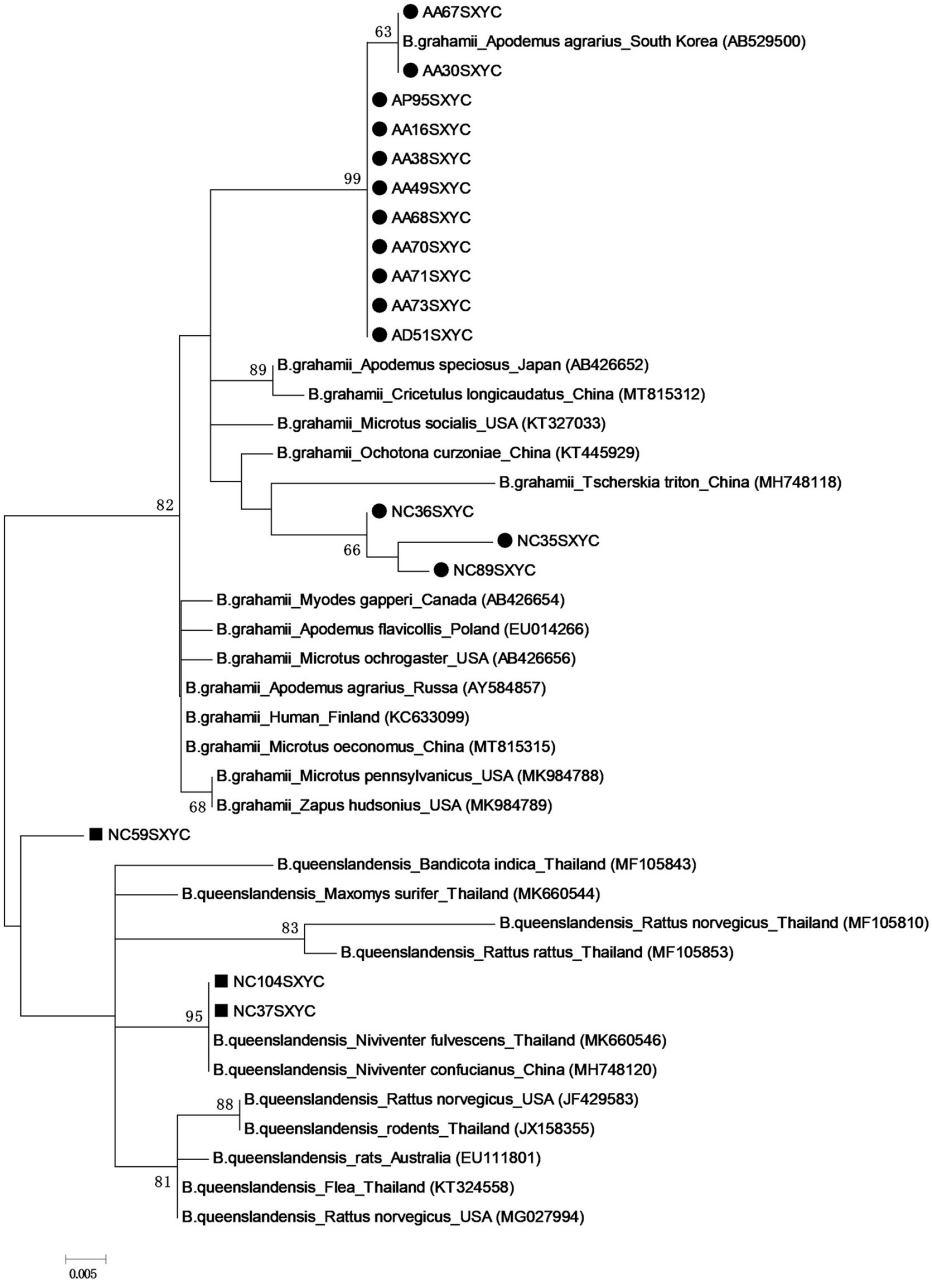
Traceability analysis of *Bartonella* species based on *gltA* gene.

We then went on to explore the likely origin of the *Bartonella* species in this area, using the *gltA* sequences of *B*. *grahamii* and *B*. *queenslandensis* placed in GenBank before July, 2021 as reference in the traceability analysis. It was shown that *B*. *grahamii* was mainly clustered into two clusters, indicating that these sequences are likely to have the different origins, with 11 of the *B*. *grahamii* sequences, including nine from *A*. *agrarius*, one from *A*. *draco* and one from *A*. *peninsulae*, shown to be most closely related to *B*. *grahamii* from *A*. *agrarius* collected in South Korea, and three *B*. *grahamii* sequences obtained from *N*. *confucianus* were most closely related to *B*. *grahamii* isolated from *T*. *triton* in China. The two *B*. *queenslandensis* sequences (NC37SXYC and NC104SXYC) obtained from *N*. *confucianus* were most closely related to *B*. *queenslandensis* from *N*. *confucianus* in China and *N*. *fulvescens* in Thailand, and one *B*. *queenslandensis* sequence (NC59SXYC) creating a unique cluster that did not correspond with any of the existing *B*. *queenslandensis* reference strains (Fig 3). This indicated that *Bartonella* infection demonstrates some specificity for specific rodent species, and that there is a high degree of the genetic diversity in the *Bartonella* species prevalent in this area.

### Genetic diversity analysis

**Fig 4:**
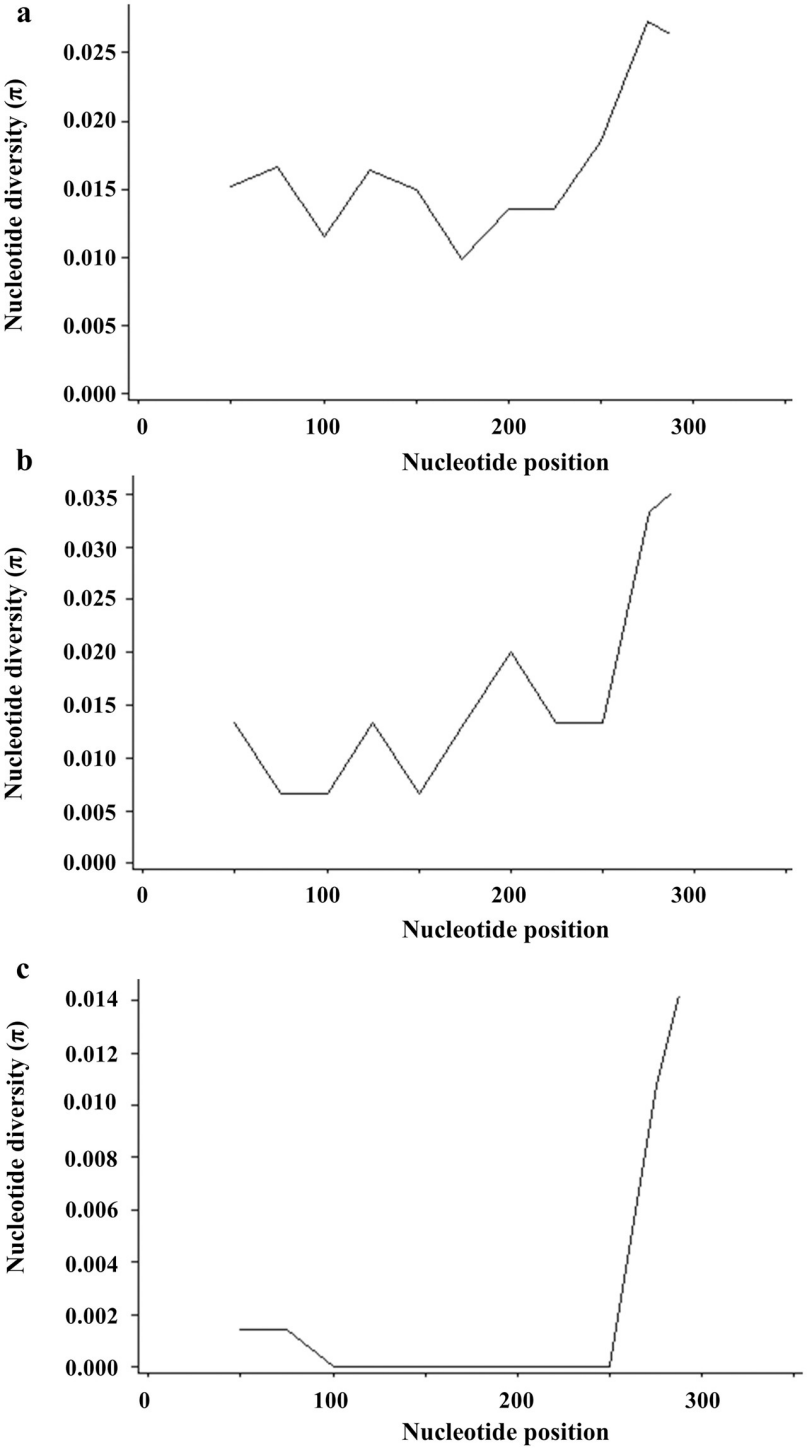
Genetic diversity of different nucleotide position in *gltA* gene of *Bartonella* species. a: *B*. *grahamii*; b: *B*. *queenslandensis*; c: *unknown Bartonella*.

Subsequently, the genetic diversity of *gltA* gene sequences (326 bp) of 14 strains of *B*. *grahamii*, 3 strains of *B*. *queenslandensis* and 14 strains of *unknown Bartonella* species revealed that the sequences from *B*. *grahamii* and *B*. *queenslandensis* exhibited noticeable intraspecies genetic diversity (π = 0.018) with the fragment diversity being highest between 275 bp and 288 bp (Table 4 and Fig 4).

**Table 4:** Genetic diversity of *Bartonella* species detected in rodents in the Zhongtiao Mountain.

*Bartonella* species (no. of sequences)	S	H	Hd (mean ± SD)	*k*	π
*B*. *grahamii* (14)	21	7	0.758 ± 0.116	5.736	0.018
*B*. *queenslandensis* (3)	9	3	1.000 ± 0.272	6.000	0.018
*unknown Bartonella* (14)	4	4	0.626 ± 0.110	1.220	0.004

S, number of polymorphic sites; H, number of haplotypes; Hd, haplotype diversity; *k*, average number of nucleotide differences; π, nucleotide diversity.

## Discussion

*Bartonella* species can cause a latent infection in their reservoirs, and this may lead to a high prevalence of *Bartonella* in the wild rodents [36]. Previous studies have shown that *Bartonella* species are widely distributed at a high frequency in wild rodents around the world. For example, the positive rate of *Bartonella* was 90.4% in *O*. *torridus* in the United States, 69% in *Apodemus* in Japan, 17% in *M*. *minutus* in Russia, and 78% in the rodents in Thailand, and so on [37, 38]. This indicates that the prevalence of *Bartonella* varies greatly in different rodents from different geographical regions.

In the present study, the prevalence and molecular characteristics of *Bartonella* species in small rodents in the Zhongtiao Mountain were observed. To our knowledge, this is the first report of the investigation in this area. Our results indicted that the infection rate of *Bartonella* species was 49.52% in small rodents, was higher than that in some areas of China, such as Zhejiang (31.4%) [28], Fujian (16.19%) [39], Yunnan (39.2%) [40], and Qinghai (18.99%, 38.61%) [41, 42], and was lower than that in Heilongjiang (57.7%) [43]. Currently, the most efficient and convenient way to detect *Bartonella* infection is by PCR. In this study, the real-time PCR targeting *ssrA* gene was used for *Bartonella* detection. The *ssrA* gene has the same species identification function as the traditional *gltA* gene, indicating that the amplification of *ssrA* fragment can be used as a fast and accurate method for the detection and classification of *Bartonella* [32]. Here we used a combination of liver, spleen and kidney tissues for *Bartonella* detection, with multi-tissue detection likely to capture a more accurate positive rate and produce as many sequences as possible for evaluation. We detected *Bartonella* species in six of the eight small rodent species identified in this study, and determined that their infection rates were significantly different. In addition, the infection rate was significantly different between different habitats, but not between genders, which was similar to the previous study [42].

DNA sequence homology and phylogenetic analyses of *gltA* gene identified three *Bartonella* species in this cohort of animals, including *B*. *grahamii*, *B*. *queenslandensis* and *unknown Bartonella* species. *B*. *grahamii* was detected in *A*. *agrarius*, *N*. *confucianus*, *A*. *draco* and *A*. *peninsulae*, which infections are primarily associated with neuroretinitis and cat scratch disease (CSD) [18, 19], suggesting *B*. *grahamii* can infect a variety of rodents, and has the ability to cause human disease in this area. In addition, *B*. *queenslandensis* was only detected in *N*. *confucianus*, and *unknown Bartonella* was only detected in *A*. *agrarius*. Unexpectedly, no *Bartonella* sequences was obtained from *E*. *inez* in this study, might be associated with the low quantity of bacteria in *E*. *inez* in this area, which still needs further investigation. These results also indicated that there is some host specificity for *Bartonella* in rodents, and that *A*. *agrarius* may be more susceptible to multiple species of *Bartonella*, which is similar to the conclusions of several other studies [43–46]. In addition, multiple *Bartonella* species can be detected in the same rodent species (e.g., *A*. *agrarius*), and one *Bartonella* specie (e.g., *B*. *grahamii*) can infect a variety of rodents, indicating that this pathogen has a strong adaptability in rodents.

Traceability analysis showed that *B*. *grahamii* was mainly clustered into two clusters, one showing close association with the isolates from South Korean *A*. *agrarius* and the other with strains from Chinese *T*. *triton* samples. Our *B*. *queenslandensis* strains were most closely related to the isolates from *N*. *confucianus* in China and *N*. *fulvescens* in Thailand. In addition, we had one *B*. *queenslandensis* sequence that did not cluster with the reported *B*. *queenslandensis* reference strains, indicating that it might be a novel genotype. In addition, genetic diversity analysis demonstrated that *B*. *grahamii* and *B*. *queenslandensis* exhibited noticeable intraspecies genetic diversity, which was similar to the results of a previous study [47].

In conclusion, we identified three species of *Bartonella*, *B*. *grahamii*, *B*. *queenslandensis* and *unknown Bartonella* in four species of rodents, *A*. *agrarius*, *N*. *confucianus*, *A*. *draco* and *A*. *peninsulae* from the Zhongtiao Mountains in China. These results also showed that *B*. *grahamii* and one potential novel *Bartonella* species were dominant in this region and that *B*. *grahamii* and *B*. *queenslandensis* had the high genetic diversity in this area. And the biological characteristics of the potential novel *Bartonella* species need to be further investigated. Our study provided a better understanding of the prevalence and molecular characteristics of *Bartonella* species in small rodents in the Zhongtiao Mountain, which could benefit prevention and control of rodent-*Bartonella* species in this area.
